# Depression symptomatology and diagnosis: discordance between patients and physicians in primary care settings

**DOI:** 10.1186/1471-2296-9-1

**Published:** 2008-01-03

**Authors:** Chizobam Ani, Mohsen Bazargan, David Hindman, Douglas Bell, Muhammad A Farooq, Lutful Akhanjee, Francis Yemofio, Richard Baker, Michael Rodriguez

**Affiliations:** 1 Family Medicine Department Charles R Drew University of Medicine and Science, 2594 Industryway, Lynwood, CA 90262, USA; 2 Family Medicine Department, Charles R Drew University of Medicine and Science, 5850 South Main Street, Los Angeles, CA 90003, USA; 3 Division of General Internal Medicine, David Geffen School of Medicine, UCLA, 911 Broxton Plaza, Los Angeles, CA 90024, USA; 4 Internal Medicine Department, Charles R Drew University of Medicine and Science, 1807 East 120th Street, Los Angeles, CA 90059, USA; 5 Research Centers in Minority Institutions Charles R Drew University of Medicine and Science, 5850 South Main Street, Los Angeles, CA 90003, USA; 6 Family Medicine Department, David Geffen School of Medicine, UCLA. 10880 Wilshire Blvd. Suite 1800, Los Angeles, CA 90024, USA

## Abstract

**Background:**

To examine the agreement between depression symptoms using an assessment tool (PHQ-9), and physician documentation of the same symptoms during a clinic visit, and then to examine how the presence of these symptoms affects depression diagnosis in primary care settings.

**Methods:**

Interviewer administered surveys and medical record reviews. A total of 304 participants were recruited from 2321 participants screened for depression at two large urban primary care community settings.

**Results:**

Of the 2321 participants screened for depression 304 were positive for depression and of these 75.3% (n = 229) were significantly depressed (PHQ-9 score ≥ 10). Of these, 31.0% were diagnosed by a physician with a depressive disorder. A total of 57.6% (n = 175) of study participants had both significant depression symptoms and functional impairment. Of these 37.7% were diagnosed by physicians as depressed. Cohen's Kappa analysis, used to determine the agreement between depression symptoms elicited using the PHQ-9 and physician documentation of these symptoms showed only slight agreement (0.001–0.101) for all depression symptoms using standard agreement rating scales. Further analysis showed that only suicidal ideation and hypersomnia or insomnia were associated with an increased likelihood of physician depression diagnosis (OR 5.41 P sig < .01 and (OR 2.02 P sig < .05 respectively). Other depression symptoms and chronic medical conditions had no affect on physician depression diagnosis.

**Conclusion:**

Two-thirds of individuals with depression are undiagnosed in primary care settings. While functional impairment increases the rate of physician diagnosis of depression, the agreement between a structured assessment and physician elicited and or documented symptoms during a clinical encounter is very low. Suicidality, hypersomnia and insomnia are associated with an increase in the rate of depression diagnosis even when physician and self report of the symptom differ. Interventions that emphasize the use of routine structured screening of primary care patients might also improve the rate of diagnosis of depression in these settings. Further studies are needed to explore depression symptom assessment during physician patient encounter in primary care settings.

## Background

Approximately 5–10% of patients attending primary care settings meet the full DSM-IV diagnostic criteria for major depression [1-3]. Additionally, about 16% of a random sample of primary care patients were estimated to have sub-syndromal depression associated with some functional impairment [4]. Numerous studies demonstrate that patients with untreated depression with co-occurring medical illnesses have higher morbidity and mortality than comparable patients who have their depression treated [5,6]. Additionally, depression is associated with marked impairment in psychosocial function, reduced productivity, increased suicide attempts, and increased health care utilization [7].

Primary care settings have become the de facto settings for the treatment of many mental health conditions, and primary care providers are often the sole contacts for more than 50% of patients with mental illness [8-10]. Minority populations utilize outpatient specialty mental health services for psychiatric symptoms and disorders at much lower rates than non-Hispanic white persons, and are more likely to receive care in general medical settings without seeing a specialist [11-15]. Efforts aimed at increasing the appropriate diagnosis and treatment of depression in minority populations have, however, met with mixed success and depression still goes under-recognized and under-treated, especially in primary care settings [16-20].

There are many factors that influence the recognition or treatment of depression in primary care settings. Some evidence suggests that depressed patients in primary care settings present often with vague somatic complaints rather than with overt complaints of depression [21,22]. In contrast, patients consulting specialty mental health providers generally must have sufficiently recognized and acknowledged their depression symptoms to seek and accept care within a specialty metal health context. Additionally, patients may avoid disclosing emotional distress to their physicians for fear of being labeled mentally ill, either because they believe their feelings are part of their medical illness or because they don't want a psychiatric diagnosis recorded in their medical record [23,24].

Physician influence on health care delivery is an increasingly important aspect of research inquiry [25-28]. Little is known about physician-patient interactions and how these affect depression diagnosis. Some evidence suggests primary care providers may have negative attitudes toward mental health problems and do not feel responsible for managing them, may lack time, consulting skills, supporting resources and may be deterred by the workload of long term treatment and monitoring. Additionally, undiagnosed depression may tend to be in patients with mild or non-functionally impaired states that further mask the emotional burden of the disease in these individuals.

While some studies have examined depression diagnosis in the context of the physician-patient encounters [29-31], others have emphasized the importance of physician training [32,33]. In 2005 Tai-Seale et al examined primary care physician's assessment of elderly patients for depression and found that in only 14% of such visits was an assessment conducted[28]. Relatively few studies have systematically examined these relationships within the context of patient self-report, physician elicitation and the influence on the agreement between the provider and the patient on the diagnosis of depression. The primary premise for this study is that the high rate of undiagnosed depression observed in primary care settings is anteceded by discordance between patients' depression symptoms and the elicitation of these symptoms by physicians.

### Study Objectives

The objective of this study was to examine the agreement between patient self-reported depression symptoms and physician documentation of these symptoms. We then examined how these self-reported symptoms predicted physician diagnosis of depression in primary care patients.

## Methods

### Study Setting

This study was conducted at two large urban outpatient primary care clinics staffed by 50 attending and resident physicians who treat primarily underserved African American and Hispanic patients. This study represents the practice patterns of all providers at both study sites.

### Design

We conducted a prospective study using interviewer administered depression assessment surveys and post clinic visit patient medical record reviews over a 1 year period. Face-to-face interviewer administered depression assessment surveys were conducted with a systematically selected sample of patients. The last patient (most recent arrival) on the waiting list for the clinic was approached for an interview. Patients consenting to participate were then screened for depression using a two-item Patient Health Questionnaire-2 (PHQ-2) [34]. Patients scoring 3 or greater were invited to participate in a more in-depth interview using the Patient Health Questionnaire-9 (PHQ-9) [35,36]. All interviews were conducted by bi-lingual staff using Spanish and English survey instruments. The patient's medical records were reviewed after the clinic visit to record physician documented depression symptoms, diagnosis, and depression care received.

Study protocols were developed, reviewed, and approved by the institutional review boards at both sites and all participants gave written informed consent.

### Study Population

Participants were eligible to participate in the study if they screened positive for depression on assessment with the PHQ-2 [34] (scored 3 or greater), had no previous diagnosis of depression, were 18 years or older, spoke English or Spanish, and consented to a review of their medical records. All participants had no previously documented diagnosis of depression in their medical record within the past 9 months prior to the interview.

### Primary Measures

The primary study outcome measure was physician elicitation and documentation of self-reported depression symptoms. Medical record reviews were conducted to record depression symptoms documented in medical record, diagnosis, and care for depression provided during the clinic visit.

### Secondary Measures

#### Depression Symptoms

Patient Health Questionnaire – 9 (PHQ-9) [35,36]. The PHQ-9 is a brief, 9-item, patient self-report depression assessment tool specifically developed for primary care settings. The PHQ-9 scores each of the 9 DSM-IV symptoms of depression through patients' self report of each symptom over a 2-week period as Not at all (score = 0), Several Days((score = 1), More than Half the Days (score = 2) and Nearly every day (score = 3), with possible total scores ranging from 0 to 27. The PHQ-9 has demonstrated acceptable reliability, validity, sensitivity, and specificity (PHQ-9 score ≥ 10 has a sensitivity of 88% and a specificity of 88% for major depression). For this study, four sub-measures of depression symptom self-report were constructed from the PHQ-9 responses based on a normal or higher threshold or cut off for responses to each question item.

#### Low symptom cutoff

PHQ-9 responses were dichotomized as symptom absent (0) or symptom present (1): PHQ-9 question items on which participants scored "0" (Not at all) were recorded as having no symptoms and scores of 1–3 were categorized as positive symptom presence.

#### High symptom cutoff

For the high symptom cutoff measure we adjusted the threshold for positive symptom presence to include only response scores of 2–3 (More than Half the Days; score = 2) and Nearly every day; score = 3) while participant responses scoring 0 and 1 (Not at all; score = 0 and Several Days; score = 1 were categorized as symptom absent.

#### Functional Impairment

Impairment resulting from depression symptoms was measured using the additional question item on the PHQ-9 (not one of the nine) which asks participants responding positively to any of the 9 question items on the PHQ-9 how difficult these symptoms have made their social, vocational and interpersonal functioning. A 4 point scale response ranging from "no difficulty at all" to "extremely difficult" was then dichotomized with participants reporting "no difficulty" categorized as having" no impairment" and participants reporting having "somewhat," "very" and "extreme" difficulty respectively categorized as having an" impairment [36]."

#### Depression Diagnosis

Depression diagnosis documented in participants medical records were also abstracted and recorded as "No depression diagnosis" or "Depression diagnosis."

#### Other Measures

Demographic characteristics of study participants were recorded. Other variables were chosen based on the literature [5-7], and their suggested association with depression. Co-occurring mental and medical conditions documented in participant medical records or self-reported by participants during the interviews occurring within the past 9 months were recorded.

### Statistical Analysis

The relationship between patient's self reported depression symptoms and physician documentation of these symptoms was examined in several ways. First, the frequency and distribution of depression symptoms, demographics, and co-occurring medical conditions were determined. Next, the level of agreement between the four sub-measures of self-reported depression symptoms and physician documentation of these symptoms were compared using Kappa statistics. We utilized the following validated interpretation of Kappa values [37]: <0 'poor', 0–0.20 'slight', 0.21–0.40 'fair', 0.41–0.60 'moderate', 0.61–0.80 'substantial', 0.81–1.00 'almost perfect' agreement. For both the low and high symptom cutoffs we also examined the independent relationship between self-reported individual depression symptoms (PHQ-9), demographic characteristics of participants, co-occurring medical conditions and depression severity (PHQ-9 scores) with the diagnosis of depression by physicians. Next, we constructed nine logistic regression models to calculate the association between the each of the self-reported symptoms (using the high cutoff) as the dependent variable and the likelihood of a depression diagnosis by physicians when controlling for demographic characteristics, co-occurring medical conditions and depression severity (PHQ-9 scores).

## Results

### Patient characteristics

A total of 2321 patients were screened for depression using the PHQ-2 [34]. Of these, 304 participants screened positive for depression and were enrolled into the study. The mean age for participants was 50.26 years and 62.8% and 24.7% were Latino and African American respectively. About 48.4% of participants had no form of health insurance (Table 1).

**Table 1:** Characteristics of Study Sample (*n *= 304)

**Variables**	**Categories**	**N**	**%**
Gender	Male	74	24.3
	Female	230	75.7
Age	≤ 34 years	29	9.5
	35–44 years	62	20.4
Mean age: **49.72 years**	45–54 years	104	34.2
Mean age: Male:** 48.05 years**	55–64 years	85	28.0
Mean age: Female: **50.26 years**	≥ 65 years	24	7.9
Ethnicity	Latino/Hispanic	191	62.8
	Black/African American	75	24.7
	Caucasian/White	20	6.6
	Other	18	5.9
Marital Status	Single/Never married	101	33.2
	Married/Living with partner	111	36.5
	Separated/Divorced	69	22.7
	Widowed	12	3.9
Employment Status	Employed full time or part time	97	38.0
	Unemployed	75	29.4
	Disabled	31	12.2
	Retired	12	4.7
	Homemaker	40	15.7
Highest Level of Educational	None	19	6.3
	Less than high school or GED	152	50.0
	High school or GED	60	19.7
	College degree or more	59	19.4
Health Insurance Status	No health insurance coverage	144	48.4
	Some source of health insurance	147	47.4
Depression Diagnosis	Depression PHQ-9 Score ≥ 10	229	75.3
	Depression Diagnosis by Physician when PHQ-9 ≥ 10	71	31.0
Depression Diagnosis and Functional Impairment	Depression PHQ-9 Score ≥ 10 and Functional Impairment	175	57.6
	Depression Diagnosis by Physician when PHQ-9 ≥ 10 and Functional Impairment	66	37.7

### Clinical Findings

#### Depression Diagnosis: Physician vs. PHQ-9

75.3% (n = 229) of study participants were depressed by PHQ-9 criteria (cutoff of ≥ 10) [36]. Of these 31.0% were diagnosed by physician as depressed. When study participants with functional impairment were selected, 57.6% (n = 175) of study participants were depressed by PHQ-9 criteria (cutoff of ≥ 10) [36], and 37.7% were diagnosed by physicians as depressed.

#### Co-occurring Medical conditions Physician Diagnosis of Depression: (Table 2)

**Table 2:** Co-occurring Medical Conditions (*n = 229*)

**Variables**		**Categories**	**N**	**%**
^a ^Medical Health Co-occurring		Hypertension	119	52.0
		Hyperlipidemia	98	42.8
		Diabetes	75	32.8
		Chronic Pain	47	20.5
		Obesity	53	23.1
		Asthma	15	6.6
		Hypothyroidism	24	10.5
		GERD ^b^	44	19.2
Mean number of co-occurring medical condition = **2.3 (std ± 1.5)**		Osteoarthritis	38	16.6
		Headaches	17	7.4
		Anxiety Disorders	14	6.1

^a ^Documented medical record diagnosis ^b ^Gastro Esophageal Reflux Disease

1 (0.3%) participant had diagnosed personality disorder

87.8% (n = 201) of the study participants meeting the PHQ-9 criteria for depression had at least one co-occurring medical condition. Hypertension was the most common condition (52%, n = 119), followed by Hyperlipidemia (42.8%, n = 98), Diabetes (32.8%, n = 75), Obesity (23.1%, n = 53), Chronic pain (20.5%, n = 47), GERD (19.2%, n = 44), Osteoarthritis (16.6%, n = 38), and Headaches (7.4%, n = 17). The mean number of chronic medical conditions was 2.3 (std ± 1.5). About 6.5% (n = 15) of the participants had an identified co-occurring mental health condition, specifically, an anxiety disorder (6.1%, n = 14).

#### Agreement between patients' self-reported depression symptoms using the PHQ-9 and Physician documentation of depression symptoms (Table 3)

**Table 3:** Agreement Between Participants Self-Reported Depression Symptoms Using The PHQ-9 And Physician Documentation Of Depression Symptoms (n = 304).

	Measure of Agreement [Kappa Coefficient] and Significance		
	
	PHQ-9 Low symptom cutoff (n)	PHQ-9 High symptom cutoff (n)	Physician symptom elicitation Frequency
Anhedonia	.002 (224)	.036 (175)	13
Depressed mood	.001 (228)	.029 (206)	27
Insomnia or hypersomnia	.018 (213)	.011 (185)	26
Feeling tired or having little energy?	.003 (224)	.017 (192)	16
Poor appetite or overeating?	.019 (192)	.047* (155)	11
Feelings of worthlessness or excessive or inappropriate guilt	.014 (182)	.030 (122)	6
Diminished ability to think or concentrate, or indecisiveness	-.011 (177)	-.032 (128)	9
Psychomotor agitation or retardation	-.009 (146)	.006 (77)	4
Recurrent thoughts of death, suicidal ideation without/with a specific plan or attempt	.027 (55)	.083 (21)	1

The agreement between depression symptoms and physician clinical assessment was between 0.001–0.101 (slight agreement). Exceptions were however observed with the agreement for the "Diminished ability to think, concentrate, or indecisiveness" symptom which showed negative correlations across depression symptom sub-measures (-.011, -.032, -.015 and -.043 respectively, indicating poor agreement). In addition, the psychomotor agitation or retardation symptom for both low symptoms cutoffs 1 and high symptom cutoff 2 also had negative kappa values (-.009 and -.014 respectively indicating poor agreement). Results from both study sites were similar and the coefficient results are indicative of practice at either study site.

#### Predictors of Depression Diagnosis by Physicians (Table 4)

**Table 4:** Independent and Adjusted association between, Physician Depression Diagnosis and PHQ-9 elicited Depression symptoms.

**Variables**	**UOR (95% CI)**	**≠ AOR (95% CI)**
Anhedonia	1.64 [0.92–2.94]	1.66 [0.90–3.05]
Depressed mood	1.44 [0.73–2.83]	1.57 [0.77–3.20]
Insomnia or hypersomnia	1.78 [0.10–3.19]	*2.02 [1.09–3.75]
Feeling tired or having little energy	1.57 [0.86–2.86]	1.57 [0.84–2.93]
Poor appetite or overeating?	*1.74 [1.02–3.00]	1.74 [0.99–3.05]
Feelings of worthlessness or excessive or inappropriate guilt	1.35 [0.80–2.29]	1.43 [0.83–2.47]
Diminished ability to think or concentrate, or indecisiveness	1.68 [0.99–2.85]	1.62 [0.94–2.80]
Psychomotor agitation or retardation	1.45 [0.81–2.61]	1.56 [0.85–2.86]
Recurrent thoughts of death, suicidal ideation without/with a specific plan or attempt	**5.47 [2.06–14.49]	**5.41 [1.92–15.25]

*Sig. at <.05 ** Sig. at <.01 † Sig. at <.001

≠: Each reported AOR represents the likelihood of depression diagnosis for each depression in nine logistic regression analysis models.

Only self-reported "suicidal ideations or thoughts", and insomnia or hypersomnia were associated with a statistically significant likelihood of depression diagnosis by physicians in participants meeting the PHQ-9 criteria for depression when we controlled for demographic characteristics, and the number of chronic medical conditions. Using the high symptom cutoff individuals with suicidal ideations or thoughts were 5 times more likely to be diagnosed with depression when compared to individuals without these symptoms. [Unadjusted OR = 5.47 (CI = 2.06–14.49) P < .01 and adjusted OR = 5.41 (CI = 1.92–15.26) P < .01]. In addition on adjustment for other independent variables, individuals with insomnia or hypersomnia were twice as likely to be diagnosed by physician with depression when compared to individuals without this symptom [OR = 2.02 (CI = 1.09–3.75) P < .05 The number of chronic medical conditions and demographic characteristics including age, gender, highest level of education and ethnicity had were not statistically significant independent predictors of physician diagnosis of depression in individuals meeting the PHQ-9 criteria for depression.

## Discussion

### Prevalence of Depression in Study Sample

Of a total of 2321 patients screened for depression using the PHQ-2 about 304 (13%) met the PHQ-2 screening criteria for a high likelihood of depression (sensitivity 83% and specificity 92%). About 75.3% (n = 229) of these enrolled study participants were depressed by PHQ-9 (>10) assessment criteria, representing about 9.8% of the total screened. The observed prevalence (9.8%) of depression in the sample is similar to most reported primary care setting estimates [1-3].

### Physician Diagnosis of Depression

This study observed a similar trend to previous studies, suggesting that depression remains under-diagnosed in most primary health care settings [16-20]. The rate of depression diagnosis by primary care providers observed in the study sample was only 31.0% in study participants meeting the PHQ-9 criteria for depression. This finding similar to the reported diagnosis rate in a recent study by Liu et al 2006 [38] though it remains lower than reported in other studies for primary care clinical settings where missed diagnosis is estimated to be approximately 50% [39-42]. There is evidence that functional impairment is strongly associated with depression [43] and increased depression severity is associated with an increase in functional impairment [36]. Additionally, the presence of functional impairment as a result of depression symptoms is a DSM-IV prerequisite for a depression diagnosis. When functional impairment was considered in addition to the PHQ-9 score as criteria for depression diagnosis, physician diagnosis rates increased to 37.7%. This finding agrees with the previous study that physician sensitivity to depression is increased when there is functional impairment [35]. However, this increase (6.7%) still allows for a missed diagnosis of depression in over 60% of depressed individuals presenting to primary care providers.

In addition to the morbidity, cost, and mortality associated with depression, we were particularly interested in this patient population because of the chronic disease profile (Table 2). Knowing that the burden and clinical outcomes from management of many of these conditions is poorer with these co-occurring conditions [5,6], we are concerned that appropriate diagnosis of depression should occur during clinical encounters with patients confronting multiple medical disorders. While the balancing of competing demands has previously been cited as a reason for physicians not exploring depressive symptoms and diagnosis during a clinic visits [44,45] the number of chronic medical conditions did not affect physician diagnosis of depression in this study (Table 4).

### Agreement between self-reported and physician documentation of depression symptoms

Across all depression symptoms the prevalent pattern of low agreement between self reported (PHQ-9) and physician elicited symptoms can be interpreted in several ways (Table 3). Specifically they include: 1) Non-documentation of symptoms by physicians: most patients with depression in primary care settings do not necessarily relate their depression symptoms as a mental health condition [21,22], The role of the physician during a clinical encounter is to elicit and relate these symptoms to a diagnosis of depression when it exists. Elicitation of symptoms of depression, however, should logically antecede an appropriate diagnosis; our study demonstrates that when self reported symptoms exists (PHQ-9), physician documentation of each individual symptom is remarkably low. It is possible to suggest that deficiency in a comprehensive assessment for depression might account for some of the observations as suggested by Tai-Seale et al 2005 [28]. 2) Non disclosure by patients of symptoms to physicians: Patients are sometimes unwilling to disclose emotional distress to their physicians for fear of being stigmatized[23,24] or are unwilling to accept a diagnosis of mental illness. While this study did not evaluate the interaction between providers and physicians during the clinic encounter, it is probable that participants disclosing depression symptoms to study interviewers will be likely to do the same with their physicians if asked. It is noteworthy to mention that the initiation and continuity of care for depression is not based on empirical biochemical markers. Current practice guidelines require empirical clinical assessment and frequent reassessments of depression symptoms as the basis for diagnosis and monitoring of treatment effectiveness. So, while physician elicitation of depression symptoms observed in this study might be suggestive of either the lack of symptom elicitation or documentation, either practice represents deficiencies in appropriate care.

### Suicidal ideations, Insomnia or Hypersomnia and Physician Depression Diagnosis

Suicide is the ninth leading cause of death in the US [46]. About 40% of individuals who successfully commit suicide had some contact with their primary care physician within a month of committing suicide [47]. While the risk for suicide in patients with mood disorders is about 15% [48], too often physicians are unaware of their patient's suicidal ideation [49]. Two findings relating to suicidal ideations in this study were 1) There was a low agreement between self reported suicidal ideations even when more stringent criteria were utilized. Secondly, even when physicians did not elicit or document suicidal ideations, the presence of these symptoms (PHQ-9) predicted a five fold increase in the likelihood of a diagnosis of depression by physicians. The increased emphasis on the recognition by physicians of suicidality in primary care patients has become an often touted tool for preventing suicides. This study suggests that physician's index of suspicion for depression increases with the presence of suicidality (PHQ-9) even when this symptom is not detected directly. Several explanations might be responsible for this observation. First, the PHQ-9 incorporates both active and passive suicidal ideation whereas primary care physicians tend to document only active suicidal ideation. Secondly, some index of distress associated with suicidality might be present in these individuals to alert physicians even when suicidality isn't recognized directly. The results showing that insomnia or hyperinsomnia is associated with an increase in physician diagnosis of depression is additionally interesting even when agreement on this symptom is low. It is suggestive that sleep disturbances promote depression assessment and diagnosis even when physicians don't document the presence of this symptom. Further studies are needed to elucidate these relationships.

## Conclusion

Two-thirds of individuals with depression are undiagnosed in primary care settings and the agreement between a structured assessment and physician elicited and or documented symptoms during a clinical encounter is very low. Interventions that emphasize the use of routine structured screening of primary care patients might also improve the rate of symptom recognition. Further studies are needed to explore depression symptom assessment during physician patient encounter in primary care settings. Limitations to the findings of this study are; the use of medical record reviews in determining physician elicitation of depression symptoms may not be representative of physician knowledge of depression symptoms, and may only reflect physician medical record keeping practices. In an attempt to address this issue, the study was conducted with over 50 attending and resident physicians. No provider was excluded from the study. Additionally, while it is assumed that patients who were willing to disclose depression symptoms to study staff would be equally likely to acknowledge these symptoms to their primary care providers, it is also possible the experience of disclosing these symptoms to the study staff immediately prior to their clinic visit was a sufficiently cathartic experience, resulting in decreased patient motivation to disclose these symptoms to their physician. In addition while physicians at both study sites have a retinue of language interpreters available for clinic encounters, language as a predictor of the observed study results was not examined by this study. Further studies to examine the influence of provider language barriers on appropriate diagnosis will be beneficial. Finally the study results may be atypical since they represent practice in only two primary care clinics.

## Competing interests

The author(s) declare that they have no competing interests.

## Authors' contributions

All authors have read and approved the final manuscript

CA: Corresponding Author

Data collection, methods and analysis

MB.

Data analysis

DH.

Data collection, background

DB

Data analysis and methods

MAF.

Study design and conclusions.

LA.

Study design and conclusions.

FY.

Methods and conclusion

RB

Data collection and methods

MR

Data collection, and methods

## Pre-publication history

The pre-publication history for this paper can be accessed here:


